# Supplementary material for: Picocyanobacteria and deep-ocean fluorescent dissolved organic matter share similar optical properties
Source: Nat Commun. 2017 May 17;8:15284. doi: 10.1038/ncomms15284 (PMC5442323; doi:10.1038/ncomms15284)
Supplement: Supplementary Information — Supplementary Figures and Supplementary Tables [file ncomms15284-s1.pdf]

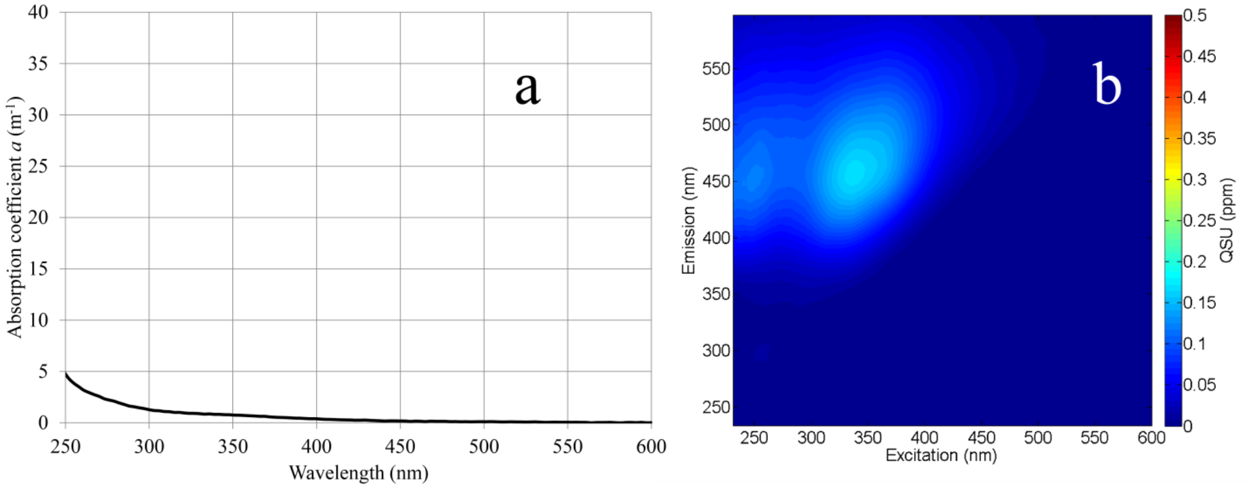

Supplementary Figure 1: UV-Vis absorption (a) and excitation emission matrix (EEM) (b) spectra of the SN medium (Cyanosite ®) used to grow *Synechococcus* cultures. Note: The same scale used in Figure 1 was also used here.

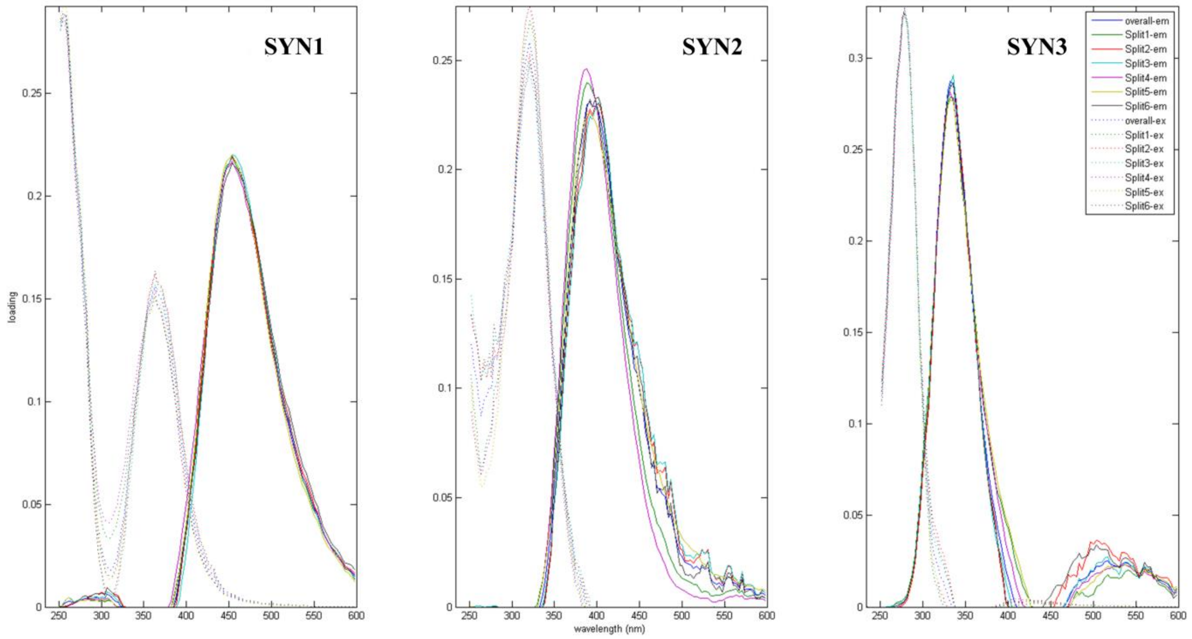

Supplementary Figure 2: Split-half validation (6 splits) of the 3-components EEM-PARAFAC model of Synechococcus (CB0101) SPE-DOM.

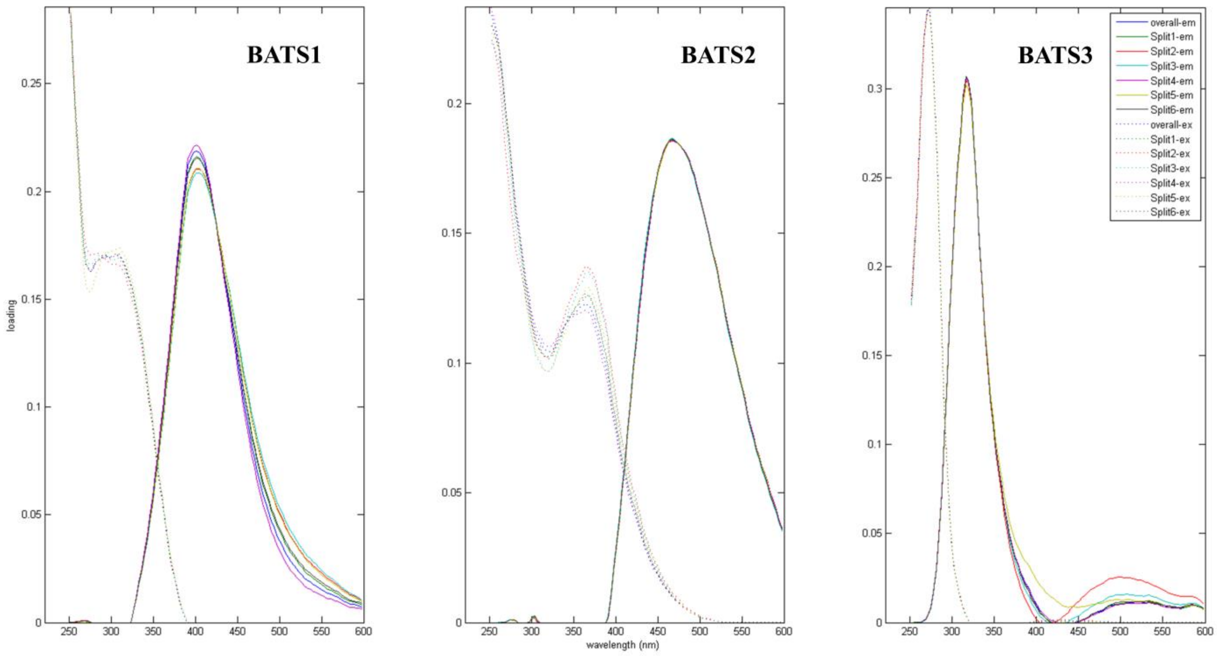

Supplementary Figure 3: Split-half validation of the 3-component EEM-PARAFAC model of the marine SPE-DOM.

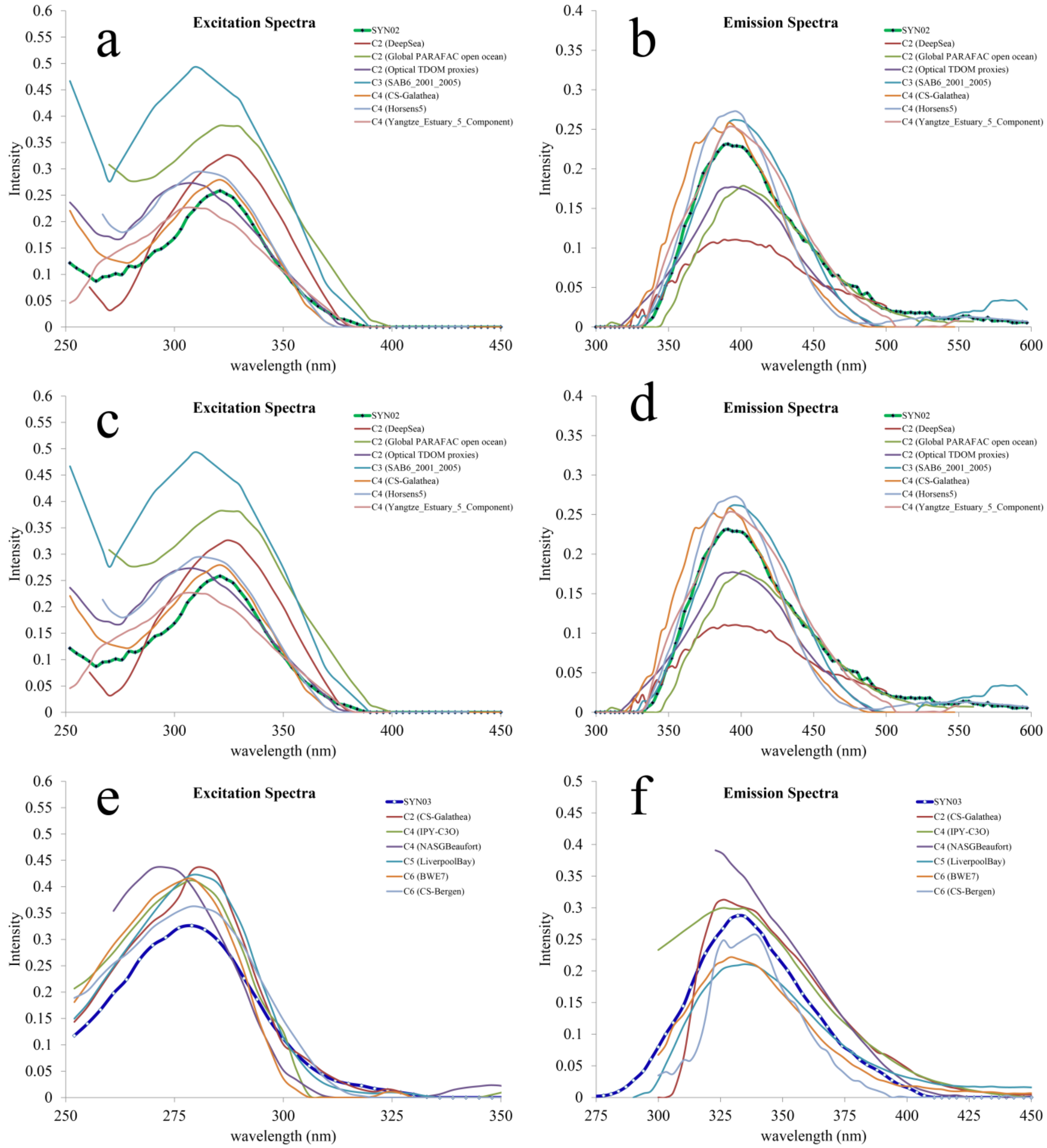

Supplementary Figure 4: Comparison of EEM-PARAFAC components of *Synechococcus* (CB0101) SPE-DOM (SYN1-3) with data sets published in Openfluor. SYN1 (a,b), SYN2 (c,d) and SYN3 (e,f).

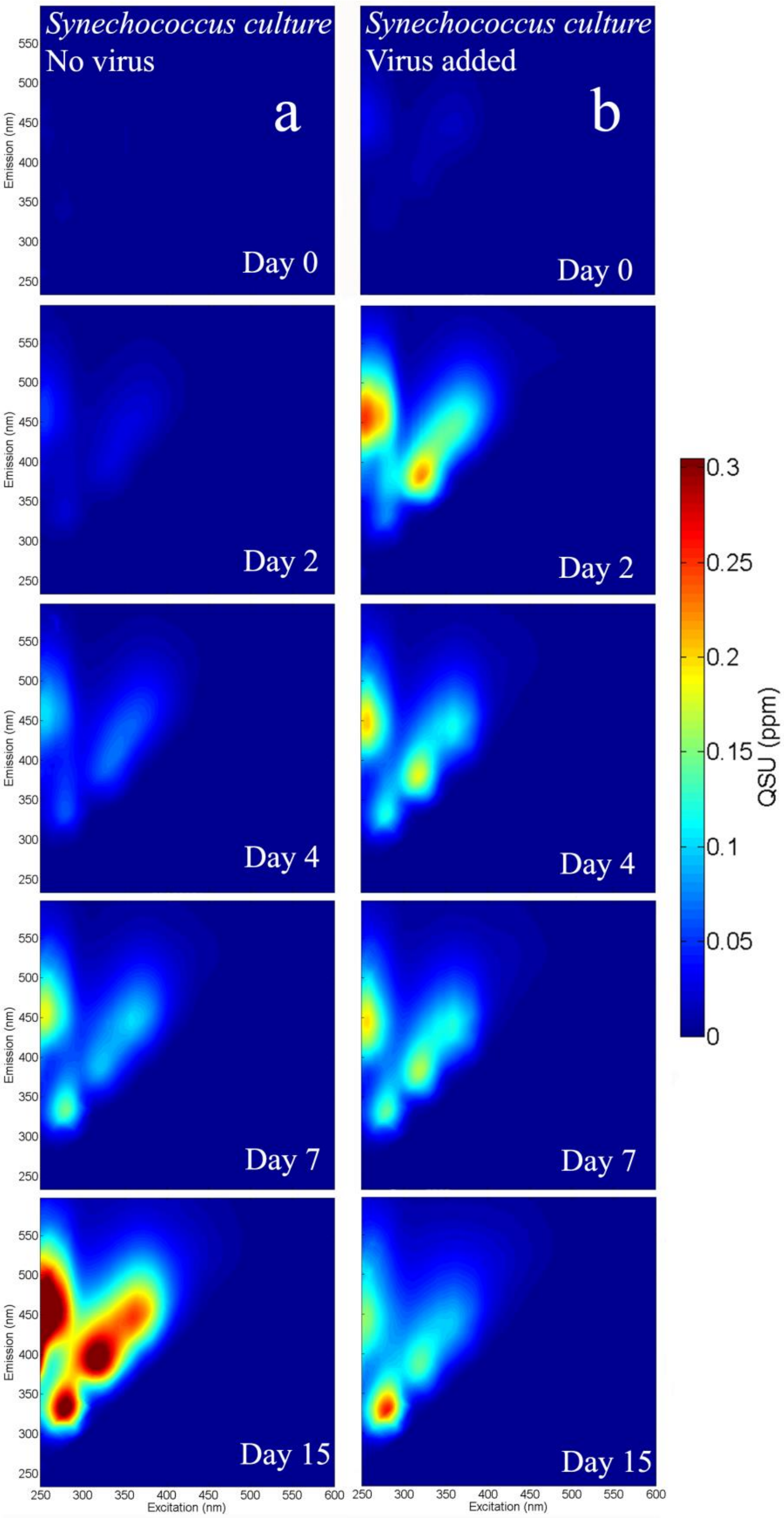

Supplementary Figure 5: Excitation emission matrix (EEM) fluorescence of *Synechococcus* (CB0101) SPE-DOM without (a) and with (b) addition of virus (*cyanophage* P1) during a 15 days incubation period.

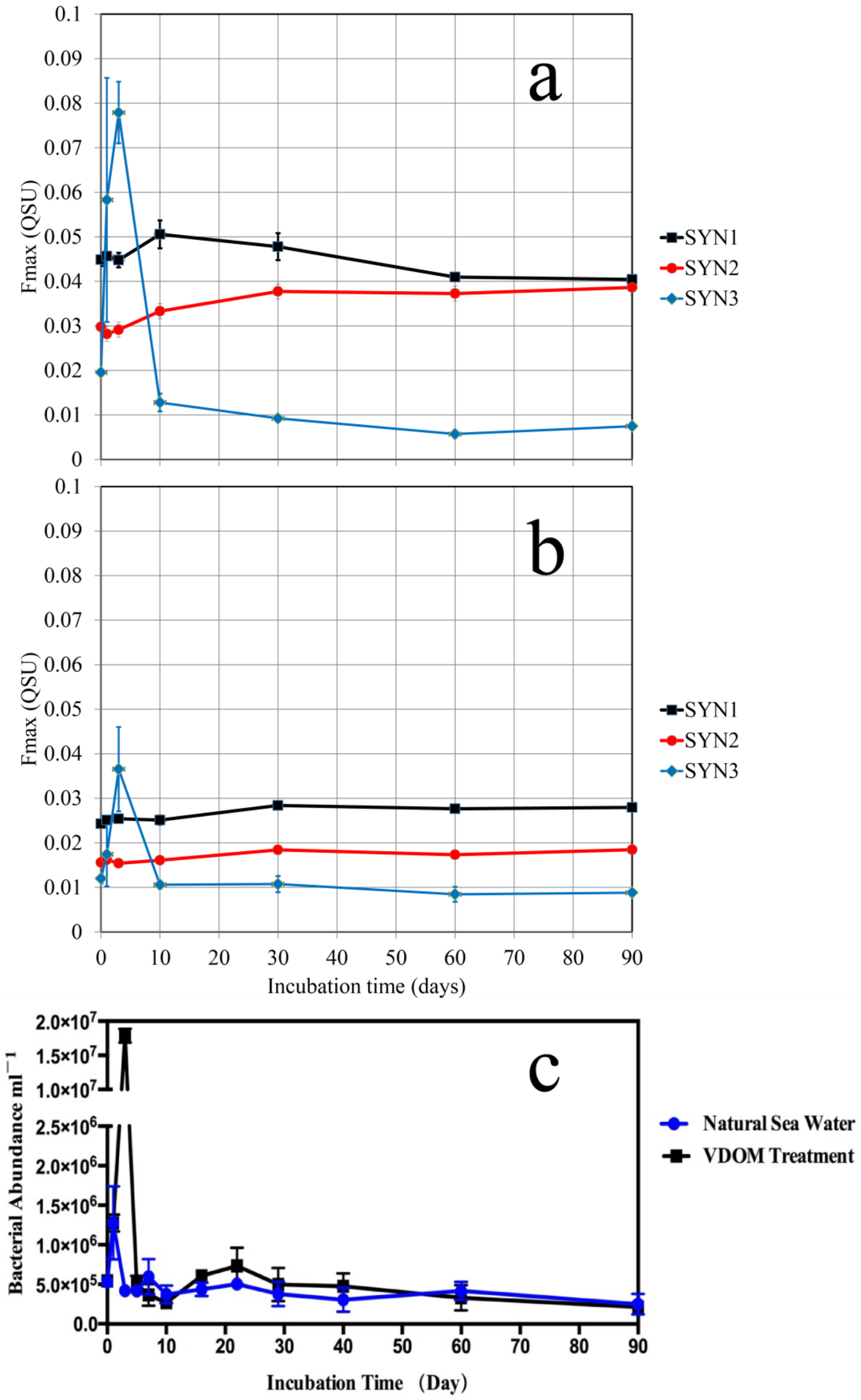

Supplementary Figure 6: Changes in SPE-DOM PARAFAC components SYN1-3 with *Synechococcus* VDOM present (a), in seawater blanks (b) and the bacterial abundance (c) during a 90 days dark incubation experiment. Note: error bars indicate the standard deviation (SD) between three replicates.

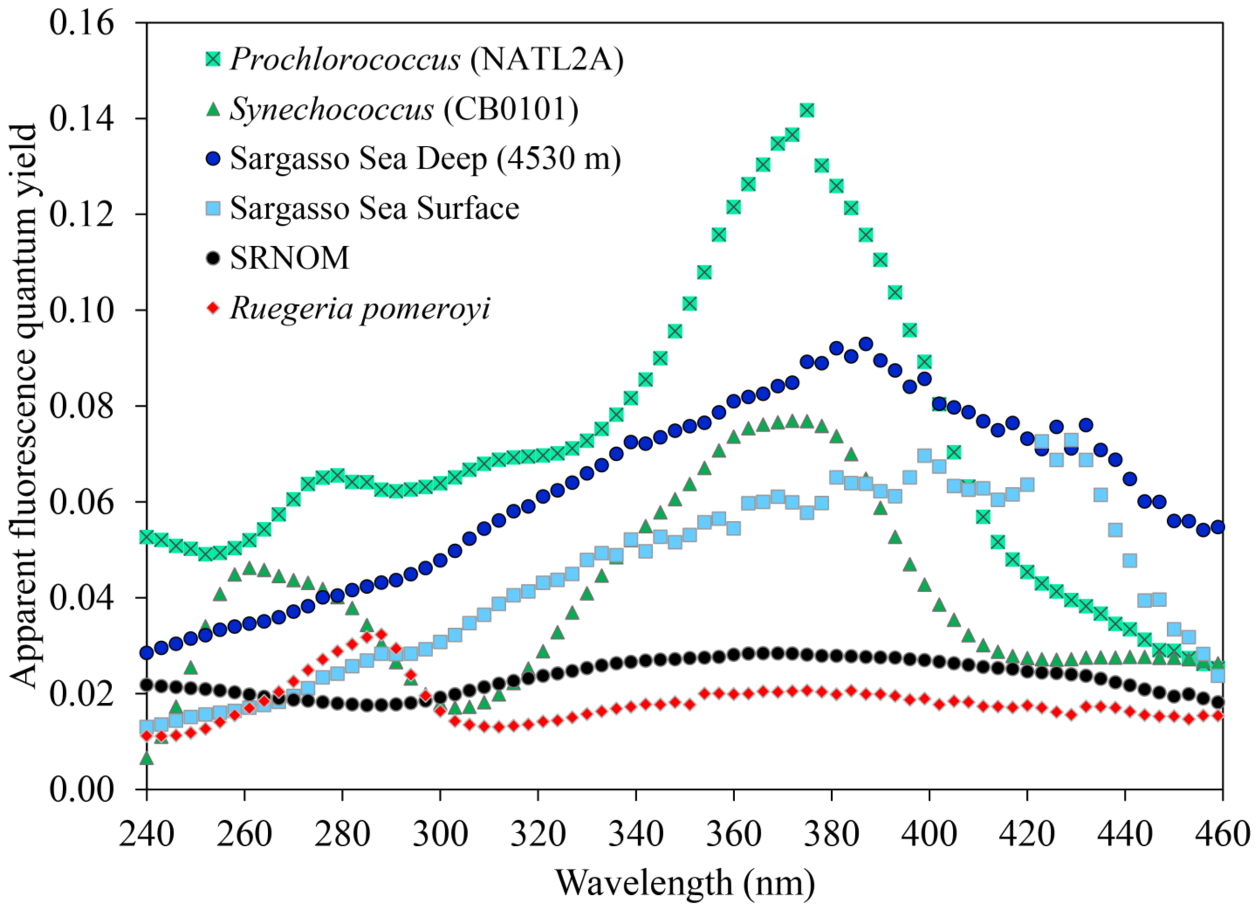

Supplementary Figure 7: Apparent fluorescent quantum yields of SPE-DOM from *Prochlorococcus* (MIT9319), *Synechococcus* (CB0101), Sargasso sea surface and deep waters, Suwannee River and the heterotrophic bacteria *Ruegeria pomeroyi* (DSS-3) normalized to the quantum yield of quinine sulfate of 0.51 (at excitation: 350 nm).

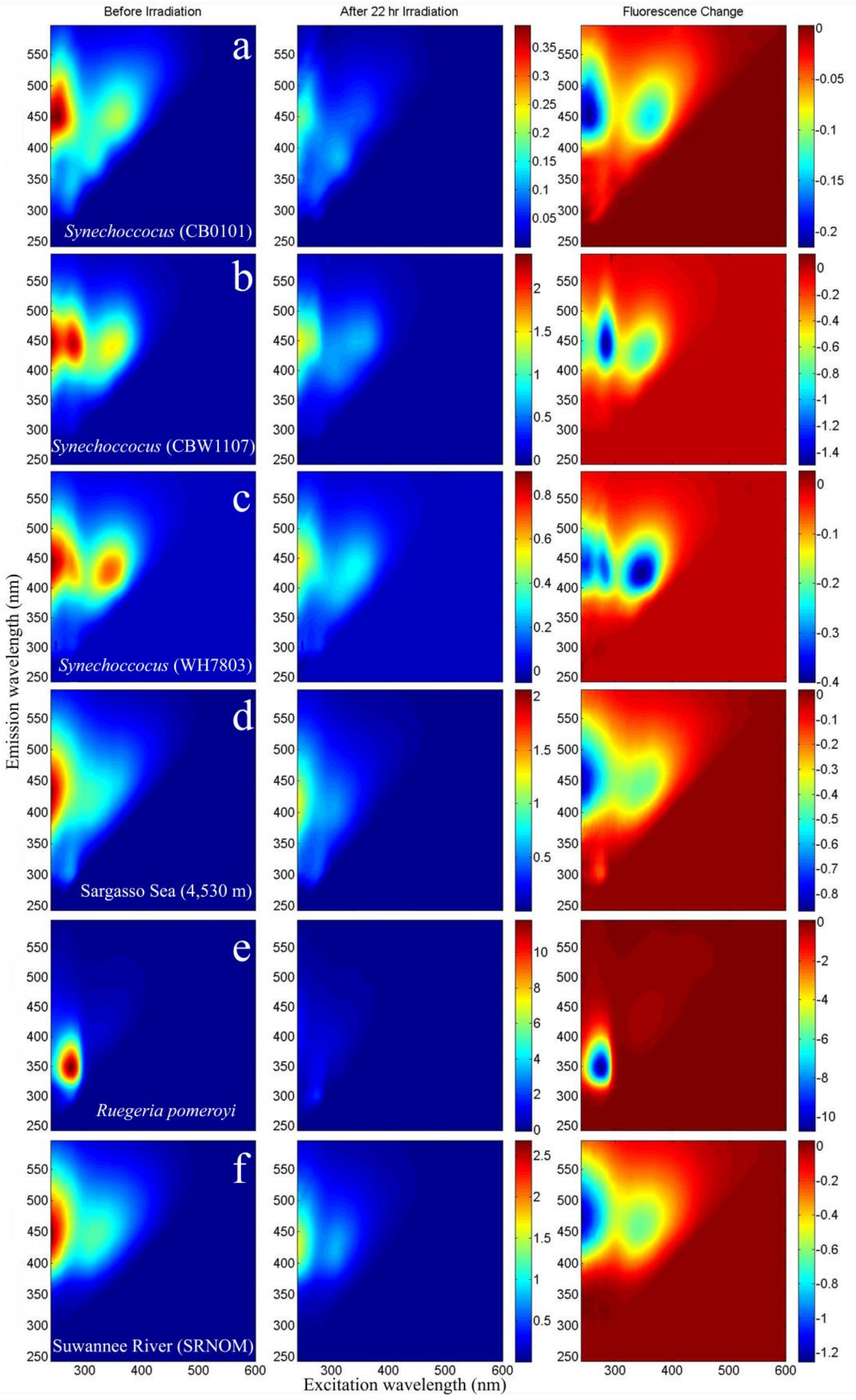

Supplementary Figure 8: Excitation Emission Matrix (EEM) spectra acquired during SPE-DOM photo-degradation of three *Synechococcus* strains ((a) CB0101, (b) CBW1107 and (c) WH7803), (d) Sargasso Sea deep-sea sample (BATS, 4530 m depth), (e) *Ruegeria pomeroyi* DSS-3 (heterotrophic bacterium) and (f) SRNOM (IHSS standard of riverine DOM).

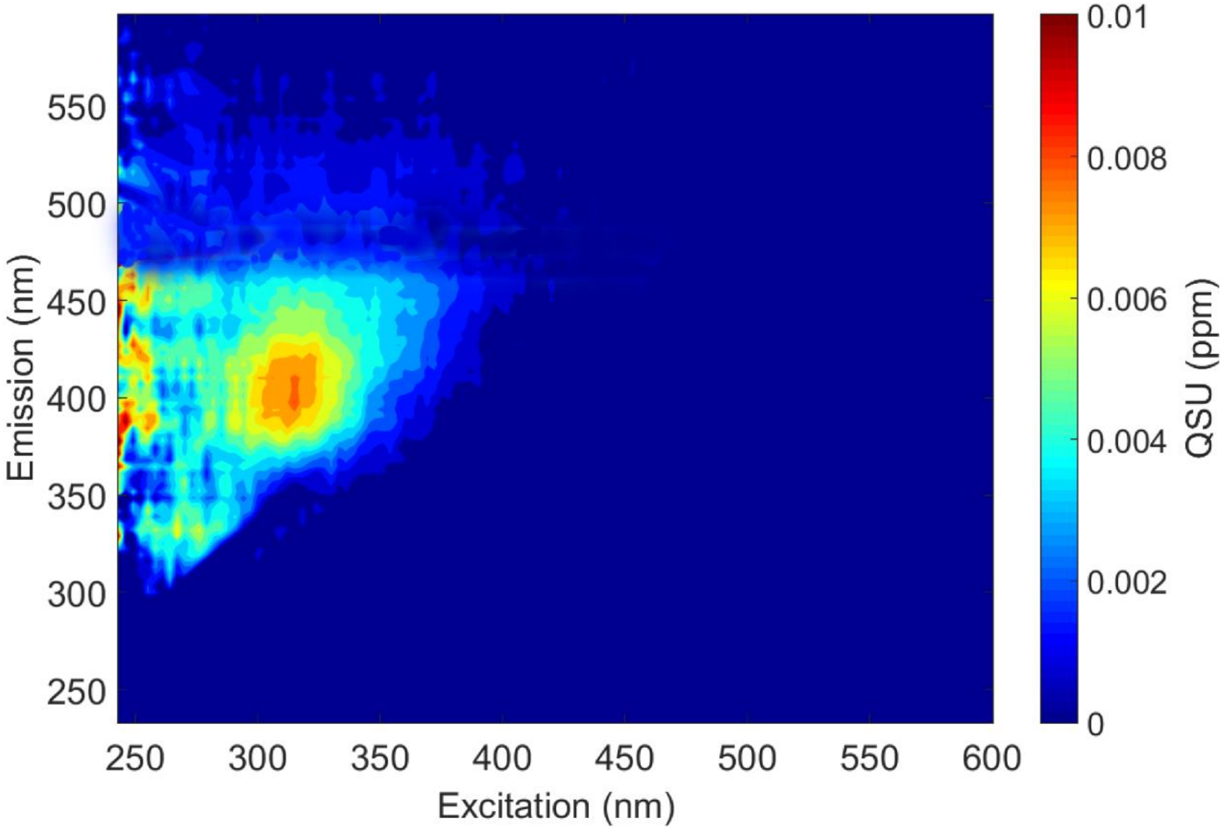

Supplementary Figure 9: Excitation emission matrix fluorescence of cultured bacteria isolated from *Synechococcus* strain (CB0101). Note: The signal is extremely weak and noise starts to show interferences at low excitation wavelengths.

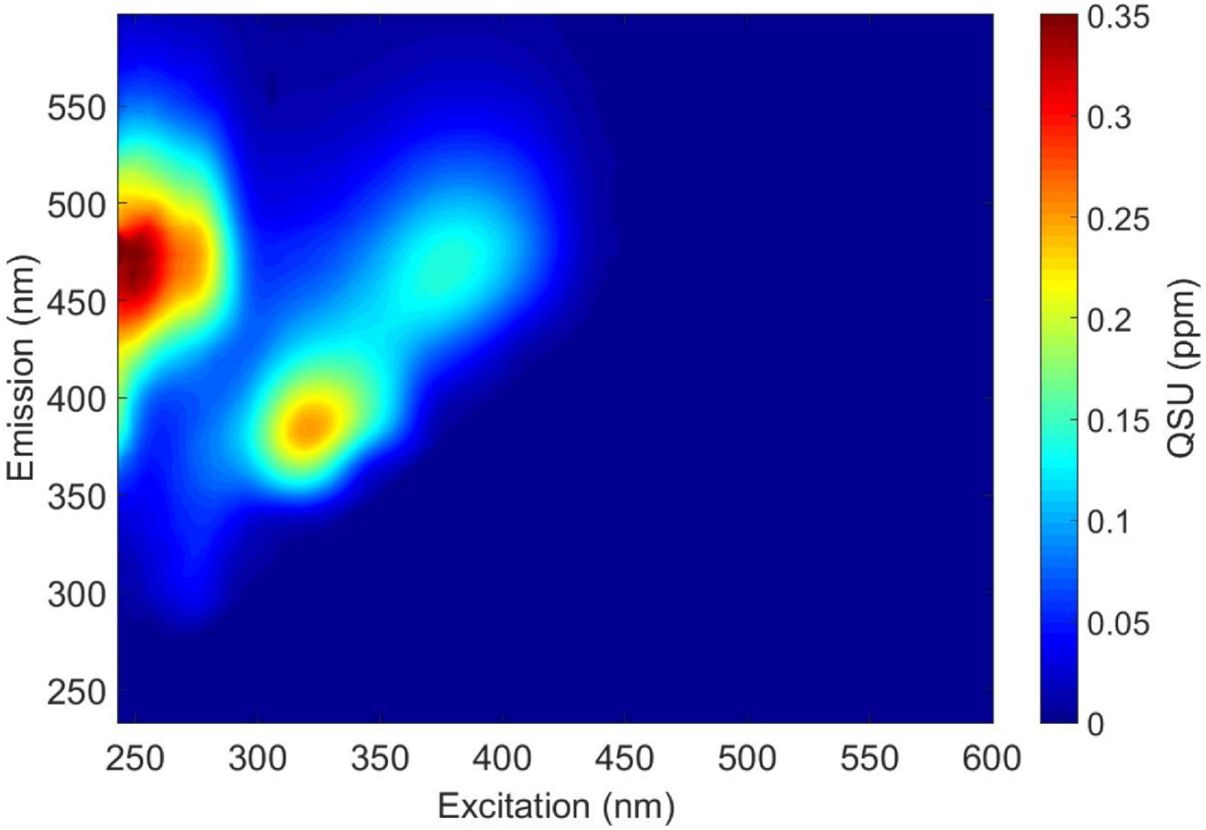

Supplementary Figure 10: SPE-DOM excitation emission matrix fluorescence of an axenic *Synechococcus* culture.

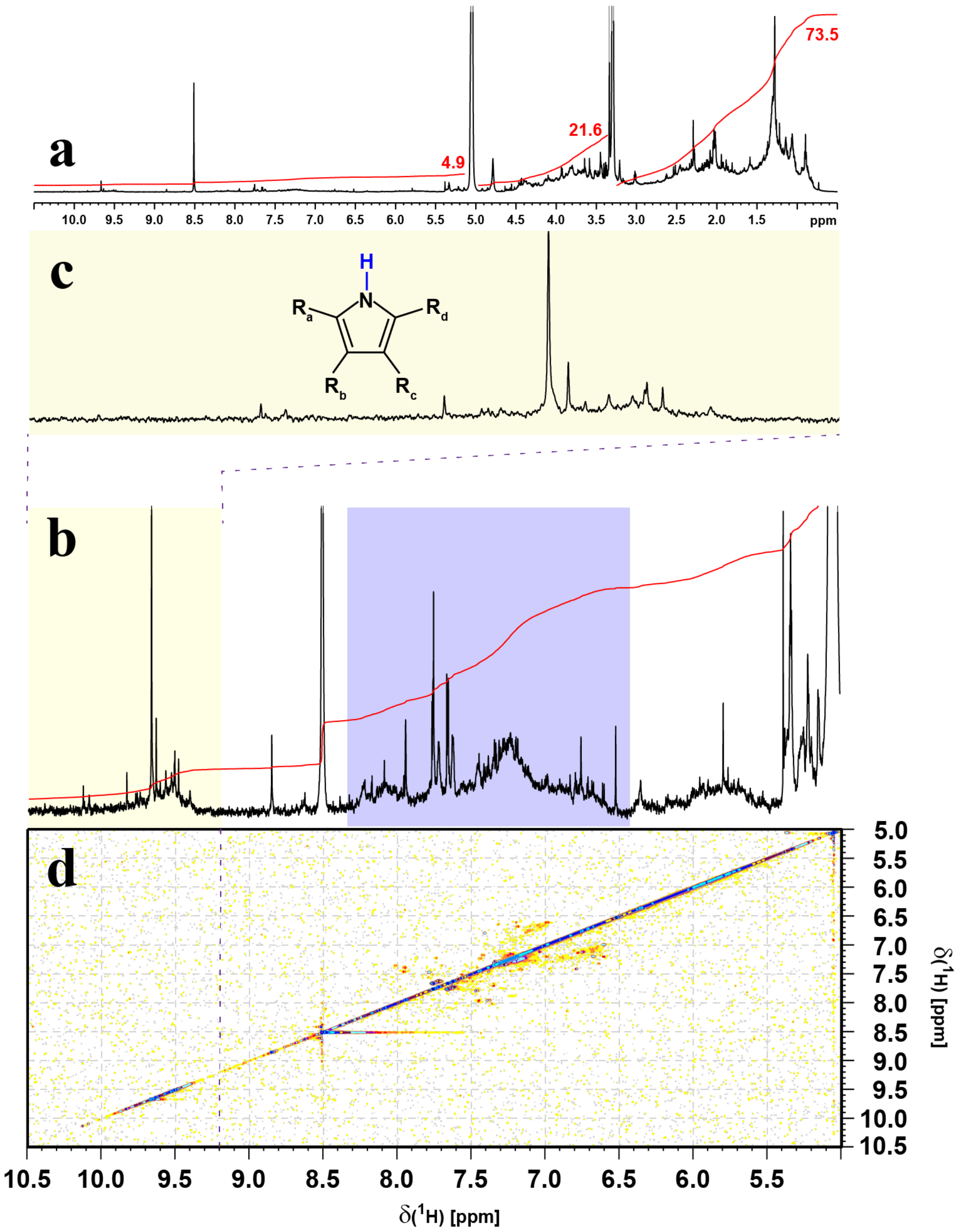

Supplementary Figure 11: of SPE-DOM; (a) full ^{1}H NMR spectrum (800 MHz, CD_3OD) of *Synechococcus* (CB0101) SPE-DOM with section integrals (see also Supplementary Table 1); (b) ^{1}H NMR aromatic section (shaded blue), and (c) the expansion of primarily pyrrolic NH resonances; (d) ^{1}H, ^{1}H TOCSY NMR spectrum of Synechococcus (CB0101) SPE-DOM. Note: the line shapes of the NH NMR resonances, which may show selective exchange broadening, deviate from the well-defined Lorentzian line shapes of common aliphatic and aromatic aldehydes which would resonate in the same section of δ_H.

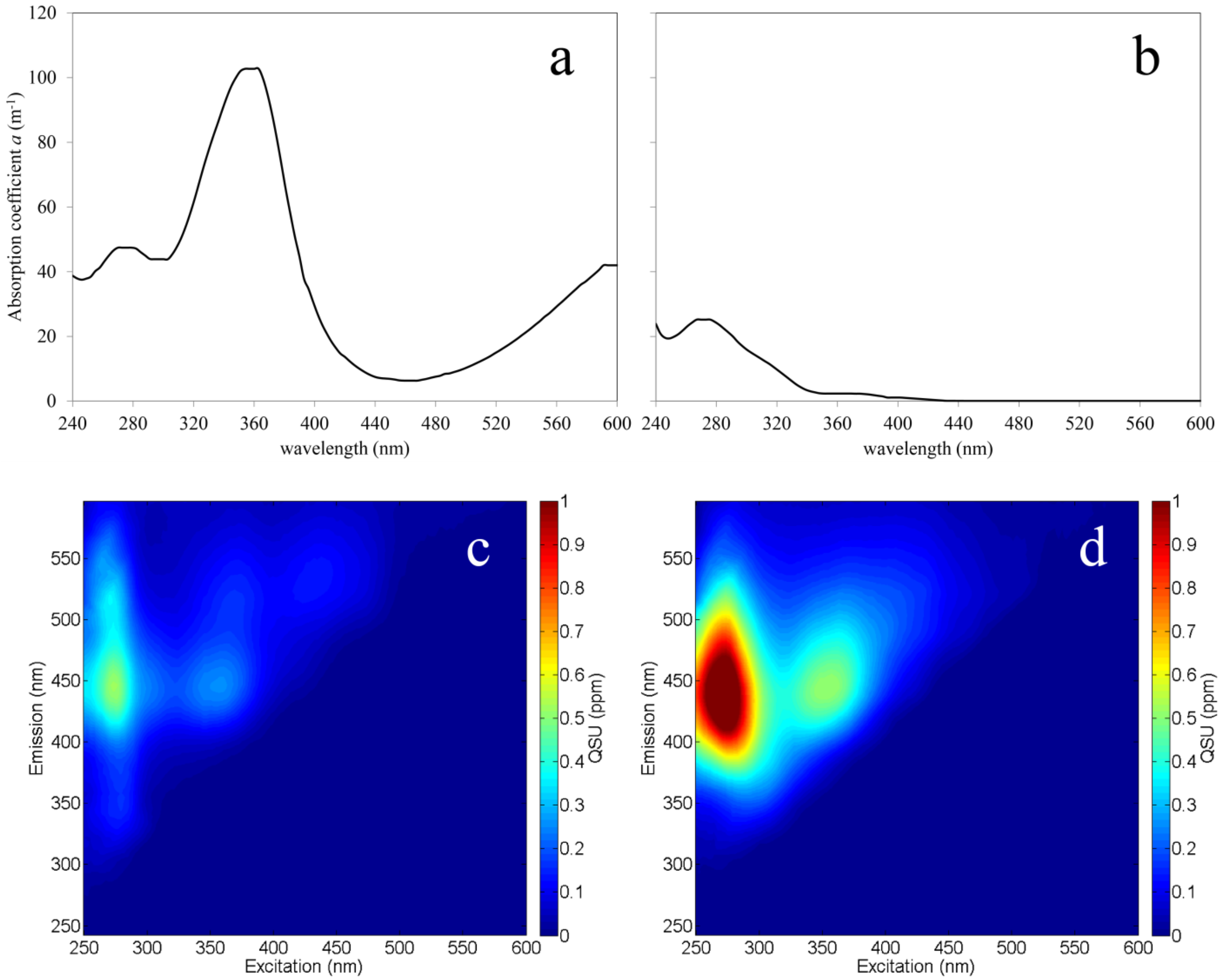

Supplementary Figure 12: UV-Vis absorption and EEM fluorescence spectra of phycocyanobilin (Frontier Scientific ®) dissolved in pure water at (a, c) 0 and (b, d) 20 hours of solar-simulated irradiance.

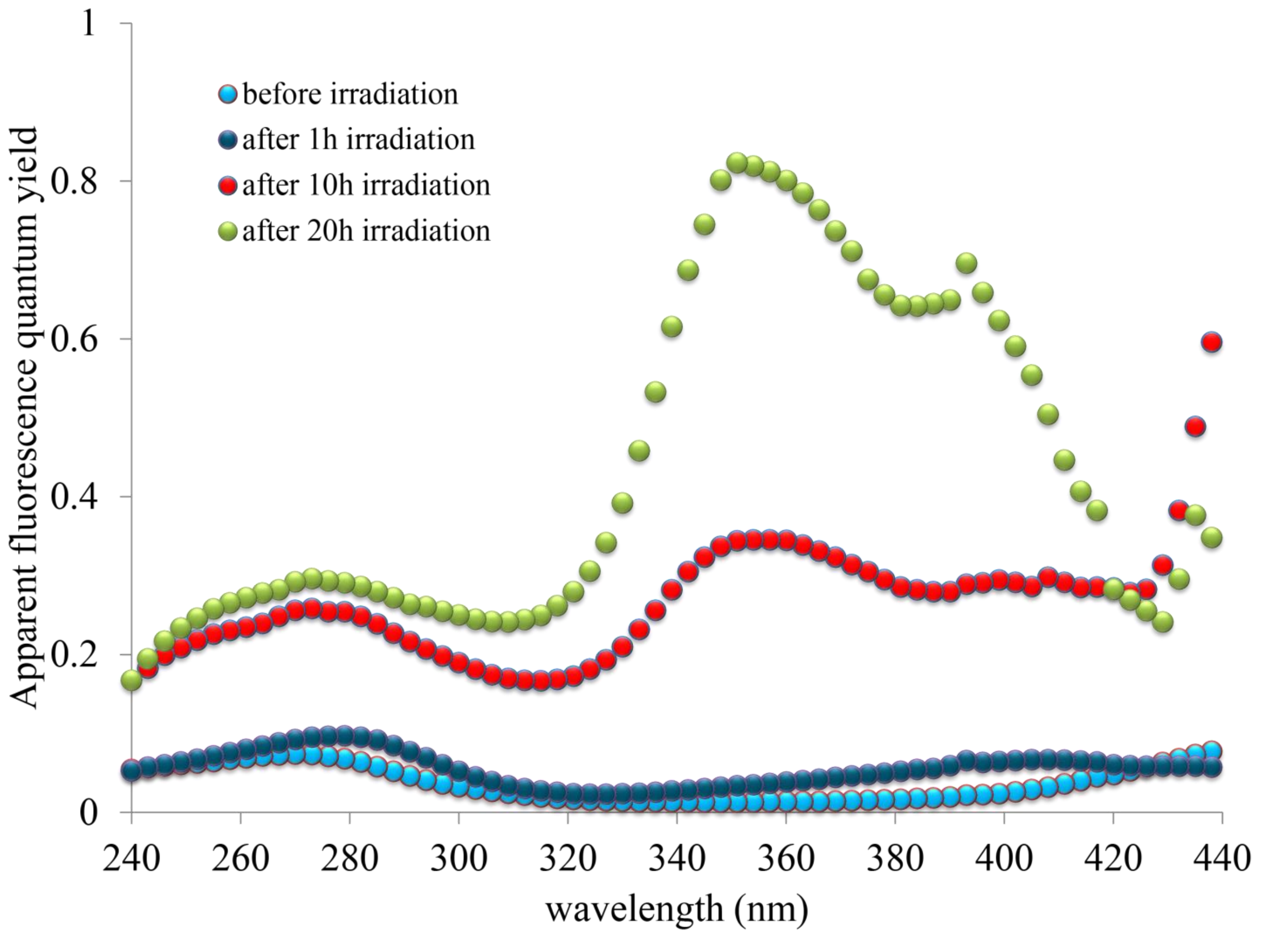

Supplementary Figure 13: Apparent fluorescence quantum yield changes during photodegradation of phycocyanobilin (Frontier Scientific ®) dissolved in pure water and normalized to the quantum yield of quinine sulfate of 0.51 (at excitation: 350 nm).

General ^{1}H NMR section integrals		
δ(^{1}H) [ppm]	probable moiety	% of sp^{2}-CH integral
10.5 – 5.2	$C_{sp2}H$	4.9
4.95 – 3.41	OCH	21.6
3.25 – 0.5	(C)CCH	73.5
Specific ^{1}H NMR section integrals between 10.5-5.2 ppm		
δ (^{1}H) [ppm]	probable moiety	% of sp^{2}-CH integral
10.5 – 9.2	pyrrole-NH	9.8
9.2 – 8.6	Six-membered N heterocycles, PAHs	1.9
8.6 – 8.4	formiate	14.4
8.4 – 7.0	aromatic rings	38.1
7.0 – 5.5	olefins (>6.5 ppm, with oxygenated aromatics)	20.1
5.5 – 5.2	CH=CH-C_{sp3} lipids and anomerics	15.6

Supplementary Table 1: ^{1}H NMR section integrals of *Synechococcus* (CB0101) SPE-DOM.

**Measured *m/z***	**Assigned neutral molecular formula**	**Neutral exact mass**	**O/C**	**H/C**	**rel. abundance**
363.11978	C17H20O7N2	364.127053	0.41	1.18	100.0
359.12486	C18H20O6N2	360.132138	0.33	1.11	58.5
377.13542	C18H22O7N2	378.142703	0.39	1.22	58.0
322.04698	C16H9O5N3	323.054222	0.31	0.56	33.2
381.09396	C16H18O9N2	382.101233	0.56	1.13	31.6
335.08848	C15H16O7N2	336.095753	0.47	1.07	29.2
375.08339	C17H16O8N2	376.090668	0.47	0.94	28.7
347.08847	C16H16O7N2	348.095753	0.44	1.00	28.2
363.08339	C16H16O8N2	364.090668	0.50	1.00	27.2
337.10413	C15H18O7N2	338.111403	0.47	1.20	26.7
365.09904	C16H18O8N2	366.106318	0.50	1.13	26.4
326.16091	C16H25O6N1	327.168189	0.38	1.56	26.4
345.10921	C17H18O6N2	346.116488	0.35	1.06	24.5
349.10412	C16H18O7N2	350.111403	0.44	1.13	24.1
374.15689	C15H25O8N3	375.164167	0.53	1.67	21.3
331.09357	C16H16O6N2	332.100838	0.38	1.00	21.3
379.07831	C16H16O9N2	380.085583	0.56	1.00	19.2
423.10453	C18H20O10N2	424.111798	0.56	1.11	19.1
319.09357	C15H16O6N2	320.100838	0.40	1.07	18.9
405.13035	C19H22O8N2	406.137618	0.42	1.16	17.5
405.09396	C18H18O9N2	406.101233	0.50	1.00	16.0
421.12527	C19H22O9N2	422.132533	0.47	1.16	15.9
361.10413	C17H18O7N2	362.111403	0.41	1.06	15.5
407.10962	C18H20O9N2	408.116883	0.50	1.11	15.2
377.09905	C17H18O8N2	378.106318	0.47	1.06	15.0
317.07792	C15H14O6N2	318.085188	0.40	0.93	14.9
351.11978	C16H20O7N2	352.127053	0.44	1.25	14.8
361.06774	C16H14O8N2	362.075018	0.50	0.88	14.4
351.0834	C15H16O8N2	352.090668	0.53	1.07	14.2
333.10922	C16H18O6N2	334.116488	0.38	1.13	13.7
321.07283	C14H14O7N2	322.080103	0.50	1.00	13.6
393.13035	C18H22O8N2	394.137618	0.44	1.22	13.6
367.11469	C16H20O8N2	368.121968	0.50	1.25	13.6
242.06698	C10H13O6N1	243.074289	0.60	1.30	13.5
403.11471	C19H20O8N2	404.121968	0.42	1.05	13.4
291.09865	C14H16O5N2	292.105923	0.36	1.14	12.8
391.1147	C18H20O8N2	392.121968	0.44	1.11	12.7
393.09395	C17H18O9N2	394.101233	0.53	1.06	12.4
391.07833	C17H16O9N2	392.085583	0.53	0.94	12.0
371.12488	C19H20O6N2	372.132138	0.32	1.05	11.8
353.09904	C15H18O8N2	354.106318	0.53	1.20	11.8
333.07282	C15H14O7N2	334.080103	0.47	0.93	11.7
375.11979	C18H20O7N2	376.127053	0.39	1.11	11.3
373.14051	C19H22O6N2	374.147788	0.32	1.16	11.3
359.08848	C17H16O7N2	360.095753	0.41	0.94	11.1
335.12487	C16H20O6N2	336.132138	0.38	1.25	10.9
403.07833	C18H16O9N2	404.085583	0.50	0.89	10.9
347.12486	C17H20O6N2	348.132138	0.35	1.18	10.6
409.08884	C17H18O10N2	410.096148	0.59	1.06	10.2

Supplementary Table 2: Nitrogen-containing molecular formula (>10 % rel. abundance) of *Synechococcus* (CB0101) SPE-DOM analyzed by negative mode electrospray FT-ICR-MS
